# Correction: Unveiling the Nexus: Influence of learning motivation on organizational performance and innovative climate of Chinese firms

**DOI:** 10.1371/journal.pone.0308062

**Published:** 2024-07-25

**Authors:** The PLOS ONE Staff

The published funding statement is incorrect. The publisher apologises for this error. The correct funding statement is: This work was supported by the Sichuan Provincial Key Research Base of Humanities and Social Sciences for Higher Education, New Construction University Reform and Development Research Centre under the project "Research on Intelligent Governance of New Undergraduate Institutions" (Project No: XJYX2021C01).

## References

[1] Zhang Y, Liao C, Liu J, Zhang Y, Gui S, Wei Q (2024) Unveiling the Nexus: Influence of learning motivation on organizational performance and innovative climate of Chinese firms. PLoS ONE 19(5): e0304729. 10.1371/journal.pone.0304729. 38820424 PMC11142492

